# Bidirectional Relationships between Adolescent Aggression and Mental Health Conditions: Longitudinal Evidence from Secondary School Students in China

**DOI:** 10.1007/s10964-025-02167-y

**Published:** 2025-03-22

**Authors:** Xiang Li, Xinxin Zhu, Rebecca P. Ang, Xintong Zhang, Yunpeng Bai, Daiyi Chen

**Affiliations:** 1 Department of Applied Social Sciences, The Hong Kong Polytechnic University, Hong Kong, China; 2 Department of Psychology, The University of Edinburgh, Edinburgh, UK; 3 Psychology and Child & Human Development Department, National Institute of Education, Nanyang Technological University, Singapore, Singapore; 4 School of Education, Trinity College Dublin, The University of Dublin, Dublin, Ireland; 5 Department of Sociology, University at Albany-SUNY, Albany, NY USA

**Keywords:** Depressive and anxious symptoms, Reactive and proactive aggression, Cyber aggression, Chinese adolescents

## Abstract

A rising global concern, adolescent aggression has been linked to adolescents’ mental health conditions, and vice versa. Although longitudinal relationships between the two have been studied, within-person associations between these variables, which are important for informing interventions, have not been adequately examined. To bridge that research gap, this study examined the within-person associations between aggression (i.e., reactive, proactive, and cyber aggression) and mental health problems (i.e., depressive and anxious symptoms), as informed by the frustration-aggression theory and the failure model. Three-wave longitudinal data were collected from a sample of Chinese adolescents (*N* = 1422; 50.9% girls; mean age = 13.56 years) at three time points, each separated by one-year intervals. The data were analyzed using a random-intercept cross-lagged panel model (RI-CLPM), revealing several within-person relationships. The presence of symptoms of depression and anxiety at T2 predicted increased cyber aggression at T3, and depressive symptoms at T2 also predicted an increase in reactive aggression at T3 (*p* < 0.1). In addition, proactive aggression at T2 predicted an increase in depressive symptoms at T3 (*p* < 0.1), and reactive aggression at T1 predicted a reduction in symptoms of anxiety at T2. All aggression- and mental health-related variables were significantly correlated at the between-person level. Moreover, the results of the multiple-group RI-CLPMs showed that gender influenced the relationships between proactive aggression and symptoms of depression and anxiety. The study’s results lend partial support to the notion of bidirectional relationships between adolescent aggressive behaviors and mental health conditions, as well as to the frustration-aggression theory and the failure model. Insights into the interactions between adolescents’ mental health problems and aggression can inform prevention and intervention strategies.

## Introduction

Adolescent aggression is a major global problem which is characterized by curvilinear patterns that peak during childhood or adolescence (e.g., Karriker-Jaffe et al., 2008). Adolescent aggression can manifest in verbal, physical, and psychological forms and can be encountered in schools and on social media platforms (Solomontos‐Kountouri & Strohmeier, 2021). Cyber aggression, involving hurtful behaviors such as sending intimidating messages and uploading offensive videos online, has emerged as a relatively new phenomenon (Ang & Goh, 2010). Scholars have divided aggression into two functional types: reactive aggression, which stems from emotional and impulsive causes, and proactive aggression, which is characterized by clear and instrumental purposes (Raine et al., 2006). Moreover, research has found a reciprocal relationship between mental health problems and aggression within the adolescent population (Zhou et al., 2024). Nevertheless, most of the relevant extant studies have focused primarily on exploring the relationships between specific mental health issues and single or limited forms of aggression and have neglected the relationship between cyber aggression and mental health problems––a limited focus that may not comprehensively support the relationship between adolescent mental health and aggression. The extent to which the reciprocal relationships between mental health problems (i.e., depressive and anxious symptoms) and different forms of adolescent aggression manifest at both the personal-trait and time-varying-state levels remains inconclusive. Furthermore, little understanding has been uncovered about gender differences in the bidirectional relationships between mental health and aggression in the existing studies, despite abundant findings that have demonstrated the existence of differences in mental health and aggression between the genders (e.g., Tokunaga, 2010; Yang et al., 2024b). In that light, this study sought to investigate the reciprocal associations between adolescent mental health conditions and reactive aggression, proactive aggression, and cyber aggression, at both the between- and within-person levels separately, and with regard to gender, among Chinese adolescents.

### Mental Health Conditions as Predictors of Aggression over Time

The frustration-aggression theory (Dollard et al., 1939), one of the most influential theories of human aggressive behaviors, argues that aggression can be elicited when individuals are limited in their ability to “reflect and process information” and no alternatives for significance restoration are available (Dollard et al., 1939, p. 452; Kruglanski et al., 2023). Concerning depression, a variety of social stressors (e.g., early childhood trauma, such as bullying and social isolation) that act as obstacles to goal achievement have been demonstrated to trigger clinical depressive symptoms among adolescents (Thapar et al., 2022). Anxiety, by definition, represents malfunctions in dealing with threats and thus is a direct consequence of the failure to achieve goals (Zorowitz et al., 2020). Furthermore, both depression and anxiety have been found to catalyze the transformation of frustrating events into aggressive behaviors (Bubier & Drabick, 2009; Fung et al., 2015).

The empirical relationships between adolescent aggression and mental health conditions have been tested (Da Silva et al., 2020). Reactive aggressors commonly display high levels of depressive and anxious symptoms, but that is not the case with proactive aggressors. For example, among early adolescents in the United States, researchers have found a positive covariation between depressive symptoms and reactive aggression, whereas they have observed a negative association between depressive symptoms and proactive aggression (Preddy et al., 2014). Notably, existing longitudinal studies have found that depressive symptoms can predict later delinquent conduct, such as physical assault (e.g., Kofler et al., 2011). Recently, a stable association was found in which anxious symptoms positively predicted indirect aggression in adolescents from ages 11 to 16 (Farrell & Vaillancourt, 2023). Moreover, a recent study conducted among Chinese early adolescents demonstrated that depression was significantly associated longitudinally with increased cyber aggression (Zhang et al., 2020). Hence, it appears to be important to focus on depression and anxiety in order to understand and prevent aggression (Thapar et al., 2022). However, the evidence is insufficient on how the symptoms of adolescent anxiety and/or depression can longitudinally predict reactive and/or proactive aggression.

### Aggression as a Predictor of Mental Health Conditions over Time

Reactive, proactive, and cyber aggression show distinct mechanisms and relevance, which have already been linked empirically to different mental health outcomes in modern contexts (Ang & Goh, 2010; Raine et al., 2006). However, findings on adolescent reactive and proactive aggressive behaviors as predictors of mental health conditions have been mixed (Burk et al., 2011; Van der Giessen et al., 2013). According to the widely applied failure model, adolescent aggressors usually encounter social rejection as a result of previous misconduct, and that in turn increases their risk of depression (Capaldi, 1992). The association between reactive-aggressive behaviors and high levels of depressive and anxious symptoms has been supported by cross-sectional studies. For example, United States adolescents with reactive-aggressive behaviors have been observed to display more anxious symptoms than proactive-aggressive adolescents did (Marsee et al., 2008). Similarly, reactive-aggressive adolescents in Hong Kong have been found to be more likely to suffer from depression and anxiety than their proactive-aggressive peers are (Fung et al., 2015). Moreover, junior high school students in Singapore who engaged in reactive aggression have been found to display high levels of anxiety, whereas such associations were not observed among proactive aggressors (Seah & Ang, 2008).

Longitudinal examinations of the failure model have been inadequate, but the existing studies do provide limited support for the model. For example, a longitudinal relationship between reactive aggressive behavior and subsequent depressive and anxious symptoms has been reported, but not between proactive-aggressive behaviors and symptoms of the two mental health conditions (Fite et al., 2014). Indeed, the presence of a relationship between reactive aggression and subsequent depression and anxiety is not surprising. Because reactive aggression is triggered primarily by uncontrollable impulses, reactive aggressors are more likely to suffer guilt and regret than proactive aggressors are, who intentionally design their aggression (Hawley & Geldhof, 2012). However, although the path from reactive aggression to later peer rejection has been supported, neither direct nor indirect longitudinal relationships have been found between reactive or proactive aggression and later depression (Evans & Fite, 2019). These insufficient findings therefore call for an in-depth examination of adolescents’ mental health conditions after they have displayed different types of aggressive behaviors.

With the emergence of the digital era, many scholars have turned their attention to instances of aggression on the internet. Studies exploring the interactive relationships between cyber aggression and mental health conditions have primarily examined how cyber aggression contributes to the victims’ impaired mental health, including their experiences of depression, anxiety, decreased life satisfaction, and suicidal ideations or attempts (Giumetti & Kowalski, 2022). Although few studies have addressed the mental health conditions of cyber aggressors, some have suggested that being a cyber aggressor is associated with increased risks of depression and social anxiety. For example, cyber aggressors were found to display a high level of social anxiety in a cross-sectional study among Spanish adolescents (Martínez-Monteagudo et al., 2020). Cyber aggressors also have been found to display higher levels of social avoidance than their non-aggressor peers did (Antipina et al., 2019). Thus, in the absence of further studies, especially longitudinal ones, the interactions between cyber aggression and mental health conditions among adolescents cannot be fully understood.

### Bidirectional Relationships between Adolescent Mental Health Conditions and Aggression

Although research on how problematic mental health states catalyze and are introduced by adolescent aggressive behaviors has generally been lacking, a few studies have initiated a discussion of the reciprocal relationships between internalizing disorders (i.e., depression and anxiety) and aggression among adolescents. For example, one review (Bubier & Drabick, 2009) concluded that anxiety in early childhood appears to precede and later contribute to disruptive behaviors, and in turn, aggression may lead to symptoms of anxiety. A more recent study employing cross-lagged panel models (CLPMs) found that anxiety and offending had a significant bidirectional relationship (Huesmann et al., 2019). Applying CLPMs to high school students in China has identified bidirectional longitudinal relationships between cyber aggression and depression (Yuan & Liu, 2021). However, researchers have raised concerns about the methodological limitations of CLPMs, arguing that they may conflate traitlike (time-invariant) and statelike (time-specific) variances and in so doing cause inaccurate inferences about dynamic within-person processes over time, obscure individual differences in these traits, and ultimately hinder the identification of cascade effects on developmental trajectories within individuals or families (Hamaker et al., 2015).

Recently, therefore, the random-intercept cross-lagged panel model (RI-CLPM) approach has been recommended for investigating longitudinal effects between psychological constructs because it can disaggregate within-person variations from between-person variations through incorporating the latent-intercept factors into the CLPM. Thus, using RI-CLPMs would be beneficial for identifying the within-person associations over time between adolescents’ levels of aggressive behavior and their depressive or anxious symptoms, and also for revealing which of the variables is causally dominant at the individual level. For instance, RI-CLPMs can assess whether a typical adolescent with higher levels of aggression than their own normal average tends to exhibit more anxious and/or depressive symptoms later on than is typical for that adolescent. Accordingly, the current study constructed RI-CLPMs to examine the evolutionary and interactive relationships between three forms of adolescent aggression and two mental health conditions. The between-person associations identified should help to delineate which groups of adolescents are more likely to show aggression or suffer from mental problems, whereas the results of the within-person modeling can suggest which aspects should be targeted for interventions to help individuals reduce their risks of aggression and/or mental problems (Masselink et al., 2018).

Nonetheless, it is important to highlight that few extant studies have tested the temporal and concurrent associations between depressive symptoms and aggression, and between anxious symptoms and aggression, especially in the context of non-Western cultures. In a collectivist society such as China’s, the priority of group harmony over individual expression can lead to internalized stress and mental health issues when individuals struggle to conform to societal expectations, and in turn that internalized stress can manifest as aggression (Zhu et al., 2021). Moreover, the Chinese value of “saving face” may cause different patterns of aggressive behaviors, because Chinese individuals often seek avoid visible conflicts in an effort to save face and maintain dignity (Xu et al., 2016). Hence, the relationship between mental health and aggression in Chinese youth might differ from that in Western societies.

Consequently, in a study involving Chinese adolescents, an RI-CLPM was performed to investigate the bidirectional cross-lagged associations between reactive and proactive aggression and depressive symptoms, with the finding that participants’ depressive symptoms showed associations with those two forms of aggression, but only at the between-person level (Yang et al., 2024a). Meanwhile, the interplay between social anxiety and aggression was examined by using an RI-CLPM, and the results revealed both that higher levels of social anxiety had robust relationships with aggression at the between-person trait level, and that there was a reciprocal predictive relationship between adolescent social anxiety and physical aggression at the within-person level (Zhou et al., 2024). However, these two studies focused primarily on a specific mental health issue or on limited forms of aggression and lacked a comprehensive consideration of the interactions between mental health problems and aggression in Chinese culture. Moreover, whereas cyber aggression has become a common public health issue worldwide, the existing literature has concentrated on traditional forms of aggressive behavior and has neglected the unique dynamics and implications of online aggression. Furthermore, the reciprocal relationship between mental health and aggression at the within-person level remains unclear, thus indicating a need for further exploration. Finally, although male and female adolescents may exhibit different aggressive behaviors and mental health conditions, these two studies lacked an in-depth examination of the gender differences in the longitudinal dynamics between mental health conditions and aggression. Overall, therefore, an understanding of the dynamic interactions between depression and/or anxiety and aggression is necessary to inform the development of culturally sensitive prevention and intervention strategies (Fung et al., 2015).

### Gender Differences

Studies have also found gender differences in the relationships between aggression and mental health. A significant relationship between social anxiety and reactive aggression was found among female undergraduate students but not among their male student peers in a cross-sectional study in the United States (Howell et al., 2015). However, other findings have contradicted the pattern and suggested that boys in a state of anxiety are more likely than girls are to engage in aggression to harm others, such as by manipulating social relationships or undermining their peers’ social status (Marsee et al., 2008). Meanwhile, in a study in China, boys were prone to exhibit a pattern of higher levels of aggression and lower levels of depression than girls did, while girls showed a depression-dominant pattern with relatively lower levels of aggression (Yang et al., 2024b). In contrast, some evidence has suggested that gender differences in both reactive and proactive aggression might not appear in clinical samples (Connor et al., 2003). Moreover, another study found that girls typically faced greater mental health risks due to cyberbullying than boys did, while boys tended to bully and be bullied by others through physical contact (Tokunaga, 2010). Given these inconsistent findings and the unique characteristics of Chinese society, more research is clearly needed.

## Current Study

Although existing research has offered direct and indirect evidence of the bidirectional relationships between mental health problems and aggression, little is understood about the longitudinal association between various types of aggression and depression and anxiety, especially when disentangling between-person effects from the varying within-person effects among Chinese adolescents. To provide a comprehensive framework for examining these relationships, the current study sought to use the RI-CLPM approach to examine the longitudinal relationships between adolescent aggressive behaviors (i.e., reactive, proactive, and cyber aggression) and mental health problems (i.e., depressive and anxious symptoms). Informed by the frustration-aggression theory and the failure model, the longitudinal relationships were expected to be bidirectional. Specifically, it was hypothesized that adolescent aggression (i.e., reactive, proactive, and cyber aggression) would predict a within-person increase in mental health problems (i.e., depressive and anxious symptoms) over time (Hypothesis 1). Likewise, it was hypothesized that two mental health problems (i.e., depressive and anxious symptoms) would predict a within-person increase in adolescent aggression (i.e., reactive, proactive, and cyber-aggression) over time (Hypothesis 2). Furthermore, given the inconsistencies in the existing findings regarding gender differences, the gender differences in the relationships between aggression and mental health were to be further explored.

## Methods

### Participants

Six secondary schools in Fujian province, China, participated in the study. Fujian province was selected to better reflect the situation of mainland China, because Fujian is neither one of the most developed nor one of the least developed areas in mainland China, according to a set of economic and social indicators (e.g., population, economic structure, and quality of life) (Li & Fung, 2015). Participants who had valid data on the variables of interest for at least one assessment time point were included in the sample. The final sample contained data from 1422 grade 7 students (50.9% girls; mean age = 13.56 years, *SD* = 0.78). Of these students, 1421 participated at Time 1 (T1), 1358 at Time 2 (T2), and 1309 at Time 3 (T3), with the consecutive time points separated by an interval of one year. The missing completely at random (MCAR) test was conducted for all of the variables over three waves of data collection and revealed a significant value for *χ*^2^ (*df*) = 749.899 (399), thus indicating that the pattern of the missing data was not a completely random one. One studied variable was not related to missingness on subsequent measurement waves, when the other studied variables were controlled for, thus indicating that the missing data were approximately equivalent to being “pure[ly] missing at random” (Newman, 2014, p. 337). Moreover, as has been suggested, attrition is expected in longitudinal studies that have many measurement points, and when that is dealt with properly it does not pose a threat to the validity of a study (Graham, 2009). Finally, the full information maximum likelihood (FIML) procedure was used to retain all of the participants who had valid data for at least one of the three time points in the analyses. The FIML approach is an ideal method that outperforms casewise and listwise deletion methods and can produce unbiased estimates even when data are not missing completely randomly (Enders & Bandalos, 2001).

### Measures

#### Reactive Aggression and Proactive Aggression

Reactive aggression and proactive aggression were measured using the Reactive–Proactive Aggression Questionnaire (RPQ; Raine et al., 2006), which has 11 items for measuring reactive aggression (e.g., “You react angrily when someone provokes you”) and 12 items for proactive aggression (e.g., “You pick a fight to show who the winner is”). The participants responded to the items using three-point Likert scales ranging from 0 (*never*) to 2 (*often*), with higher scores reflecting higher levels of reactive aggression or proactive aggression. The RPQ was previously validated among Chinese adolescents (Li & Fung, 2015). In the present study, the Cronbach’s α values for reactive aggression were 0.85, 0.84, and 0.87 for T1, T2, and T3, respectively, and the Cronbach’s α values for proactive aggression were 0.88, 0.87, and 0.91 for T1, T2, and T3, respectively.

#### Cyber Aggression

Cyber aggression was measured via the Cyberbullying Questionnaire (Ang & Goh, 2010), which consists of nine items (e.g., “I purposely excluded someone from the discussion group”). The participants responded to the items using five-point Likert scales ranging from 1 (*never*) to 5 (*a few times every week*), with higher scores indicating higher levels of involvement in cyber aggression. This scale has been validated for use in the Chinese culture, with good psychometric properties (e.g., Chan & Wong, 2019). In the present study, the scale showed good reliability, with Cronbach’s α values of 0.81, 0.83, and 0.90 for T1, T2, and T3, respectively.

#### Depressive Symptoms

Depressive symptoms were measured using the short version of the Center for Epidemiologic Studies Depression Scale (CES-D; Andresen et al., 1994), which consists of 10 items (e.g., “I felt depressed”). The participants rated the items on four-point Likert scales ranging from 0 (*rarely*) to 4 (*all the time*), with higher scores indicating higher levels of depression. The CES-D has been validated to perform reliably among Chinese adolescents (e.g., Wang et al., 2021). In the present study, the scale had good reliability, with Cronbach’s α values of 0.79, 0.82, and 0.81 for T1, T2, and T3, respectively.

#### Anxious Symptoms

To measure anxious symptoms, the anxiety subscale of the Hospital Anxiety and Depression Scale was used (HADS; Zigmond & Snaith, 1983). The subscale consists of seven items (e.g., “My mind was full of worrying thoughts”), and the participants responded to the items using four-point Likert scales ranging from 0 (*never*) to 3 (*most of the time*), with higher scores indicating higher levels of anxious symptoms. The HADS has shown good psychometric properties in community samples, especially in China (Chan et al., 2010). The scale had good reliability in the present study, with Cronbach’s α values of 0.84, 0.86, and 0.86 for T1, T2, and T3, respectively.

### Data Analysis Plan

First, descriptive statistics and correlations were calculated using SPSS 29.0 software, and the longitudinal measurement invariances of reactive aggression, proactive aggression, and cyber aggression, and of depressive symptoms and anxious symptoms, were examined through confirmatory factor analysis (CFA). In addition, the measurement invariances were tested for the measures at Time 1 across genders. Model comparison results with a change in the values for the Comparative Fit Index (CFI) < 0.010, combined with a change in values for the Residual Root Mean Square Error of Approximation (RMSEA) < 0.015 or the Standardized Root Mean Square Residual (SRMR) < 0.030, supported the measurement invariance.

Second, the intraclass correlations (ICCs) for the studied variables were calculated to identify the amount of variance that was described by the stable differences between people, instead of the variance caused by fluctuations across time within individuals. Then, RI-CLPMs were used to test the hypotheses, because RI-CLPM models can assess within-person processes separately from between-person ones (Hamaker et al., 2015). Although traditional CLPMs are considered useful for drawing causal inferences, recent methodological innovations have challenged their validity, as mentioned above. Specifically, autoregressive parameters in CLPMs cannot control for time-invariant constructs––in other words, within-person effects cannot be distinguished from between-person effects in traditional CLPMs (Hamaker et al., 2015). To overcome that problem, those authors proposed RI-CLPMs, which incorporate random intercepts to account for the compounding effects arising from traitlike stability and which allow the intercepts to covary in order to capture the overall stability of the relationships between constructs at the between-person level. The RI-CLPMs do not blend different sources of variability, and they prevent “erroneous conclusions regarding the presence, predominance, and sign of causal influences” (Hamaker et al., 2015, p. 102). The autoregressive paths in an RI-CLPM represent within-person changes over time, thus indicating, in this study, that a juvenile’s aggression and mental health levels at one time may differ from those at another time. The cross-lagged paths captured within-person reciprocal associations between variables.

To reduce the complexity of the modeling, six separate models were computed for the relationships between each type of aggressive behavior and the symptoms indicating two mental health issues: a model for reactive aggression–depressive symptoms (M1), a model for reactive aggression–anxious symptoms (M2), one for proactive aggression–depressive symptoms (M3), one for proactive aggression–anxious symptoms (M4), one for cyber aggression–depressive symptoms (M5), and a model for cyber aggression–anxious symptoms (M6). Gender was specified as a time-invariant covariate at the between-person level (in each model). The between-person variances were captured by two random intercepts, one consisting of the three observed scores of aggression and the other consisting of the three observed scores of depressive symptoms and anxious symptoms, while the within-person variances were captured by latent factors composed of each observed score of aggression and depressive symptoms or anxious symptoms regressed on its own latent factor, with factor loadings constrained to 1. These latent factors represented the within-person changes around an individual’s expected (mean) score. Moreover, multiple-group RI-CLPMs were performed to investigate the moderating effects of gender on the reciprocal relationships between aggression and mental health, by comparing the models in which cross-lagged paths coefficients were constrained with the models in which the paths were not constrained. A nonsignificant chi-square difference between the compared models indicated that the lagged effects of different levels of aggression or mental health were the same between males and females. Because the multiple linear regression technique cannot be used for chi-square difference testing regularly, the Satorra-Bentler scaled Chi-Square difference test was used (Satorra & Bentler, 2010). All modeling was performed in Mplus 8.8 with the maximum likelihood estimator (MLR). Values greater than 0.90 on the CFI and the TLI, and values smaller than 0.06 and 0.08 for the RMSEA and SRMR, respectively, were considered acceptable.

## Results

### Descriptive Statistics

The means, standard deviations, and correlations between the five key variables are presented in Table 1. Across the three time points, significant positive correlations were observed between the various types of aggression and both depressive symptoms and anxious symptoms, except for depressive symptoms at T1 and cyber aggression at T3. In addition, this study measured the longitudinal invariances across the waves of data collection, including configural, metric, and scalar invariances. As is displayed in Table 2, the configural, metric, and scalar invariances of all measures were established, thus suggesting that the variables of interest had measurement invariances over time. Meanwhile, gender measurement invariance was also tested for the measures at T1, and all measures (except the RPQ) provided configural, metric, and scalar invariance. Metric measurement invariance was established for the RPQ at T1.

**Table 1:** Descriptive statistics and bivariate correlations for study variables

	*M*	*SD*	T1_CA	T2_CA	T3_CA	T1_RA	T2_RA	T3_RA	T1_PA	T2_PA	T3_PA	T1_DE	T2_DE	T3_DE	T1_Anx	T2_Anx	T3_Anx
T1_CA	9.842	2.419	_														
T2_CA	10.008	2.557	0.290***	_													
T3_CA	10.013	3.192	0.210***	0.286***	_												
T1_RA	4.357	3.851	0.302***	0.188***	0.088**	_											
T2_RA	4.747	3.903	0.153***	0.276***	0.193***	0.471***	_										
T3_RA	3.868	3.828	0.147***	0.162***	0.334***	0.372***	0.495***	_									
T1_PA	1.155	2.569	0.340***	0.235***	0.126***	0.584***	0.243***	0.193***	_								
T2_PA	1.204	2.555	0.154***	0.424***	0.283***	0.257***	0.549***	0.279***	0.338***	_							
T3_PA	1.037	2.556	0.184***	0.180***	0.532***	0.140***	0.222***	0.578***	0.199***	0.358***	_						
T1_DE	7.973	5.376	0.188***	0.125***	0.044	0.331***	0.235***	0.218***	0.219***	0.125***	0.086**	_					
T2_DE	8.937	5.641	0.092**	0.156***	0.097***	0.226***	0.318***	0.272***	0.125***	0.189***	0.080**	0.479***	_				
T3_DE	9.085	5.473	0.097***	0.106***	0.140***	0.205***	0.262***	0.397***	0.141***	0.172***	0.234***	0.416***	0.491***	_			
T1_Anx	6.725	4.351	0.158***	0.120***	0.071*	0.331***	0.268***	0.278***	0.182***	0.124***	0.126***	0.656***	0.429***	0.368***	_		
T2_Anx	6.860	4.438	0.095***	0.125***	0.120***	0.178***	0.332***	0.274***	0.102***	0.163***	0.113***	0.397***	0.677***	0.443***	0.464***	_	
T3_Anx	6.738	4.351	0.064*	0.117***	0.123***	0.206***	0.283***	0.400***	0.117***	0.173***	0.176***	0.353***	0.465***	0.706***	0.419***	0.509***	_

*CA* cyber-aggression, *RA* reactive aggression, *PA* proactive aggression, *DE* depression, *Anx* anxiety

*p < 0.05, **p < 0.01, ***p < 0.001

**Table 2:** Measurement invariance

		*χ*^*2*^(*df*)	CFI	TLI	RMSEA	SRMR	AIC	BIC	△CFI	△RMSEA
**Longitudinal**										
RPQ	Configural	3493.232(2056)	0.938	0.930	0.022[0.021, 0.023]	0.055	53710.025	55961.227		
	Metric	3532.753(2098)	0.939	0.931	0.022[0.021, 0.023]	0.056	53777.921	55808.212	0.001	0.000
	Scalar	3635.087(2140)	0.936	0.930	0.022[0.021, 0.023]	0.056	53815.689	55625.067	−0.003	0.000
HADS	Configural	590.403(165)	0.966	0.957	0.043[0.039, 0.046]	0.041	55200.778	55658.382		
	Metric	609.282(177)	0.965	0.959	0.041[0.038, 0.045]	0.043	55194.764	55589.250	−0.001	−0.002
	Scalar	683.951(189)	0.960	0.956	0.043[0.039, 0.046]	0.045	55254.073	55585.441	−0.005	0.002
CES-D	Configural	850.600(334)	0.966	0.956	0.033[0.030, 0.036]	0.051	87861.481	88708.312		
	Metric	905.770(352)	0.964	0.955	0.033[0.031, 0.036]	0.055	87889.139	88641.293	−0.002	0.000
	Scalar	989.445(370)	0.960	0.952	0.034[0.032, 0.037]	0.055	87942.174	88599.652	−0.004	0.001
CA	Configural	473.080(276)	0.949	0.935	0.022[0.019, 0.026]	0.057	23225.532	23904.049		
	Metric	477.823(292)	0.952	0.942	0.021[0.018, 0.025]	0.067	23371.926	23966.286	0.003	−0.001
	Scalar	504.509(308)	0.949	0.942	0.021[0.018, 0.024]	0.066	23366.479	23876.681	−0.003	0.000
**Gender**										
RPQ	Configural	567.233(336)	0.958	0.936	0.031[0.027, 0.036]	0.049	20074.870	21452.205		
	Metric	627.292(357)	0.950	0.930	0.033[0.028, 0.037]	0.068	20215.425	21482.362	−0.008	0.002
	Scalar	757.046(378)	0.930	0.907	0.038[0.034, 0.041]	0.071	20406.849	21563.390	−0.020	0.005
HADS	Configural	35.060(16)	0.994	0.983	0.041[0.022, 0.060]	0.016	20134.272	20417.653		
	Metric	59.342(29)	0.990	0.985	0.039[0.024, 0.053]	0.046	20144.088	20359.248	−0.004	−0.002
	Scalar	77.985(35)	0.985	0.982	0.042[0.029, 0.054]	0.048	20153.141	20336.814	−0.005	0.003
CES-D	Configural	88.068(48)	0.988	0.978	0.034[0.023, 0.046]	0.026	31945.054	32375.780		
	Metric	137.802(69)	0.979	0.973	0.038[0.028, 0.047]	0.052	31976.306	32296.724	−0.009	0.004
	Scalar	161.137(78)	0.975	0.971	0.039[0.030, 0.047]	0.052	31983.512	32256.656	−0.004	0.001
CA	Configural	64.204(38)	0.963	0.931	0.031[0.017, 0.044]	0.045	6354.309	6722.447		
	Metric	76.900(54)	0.968	0.957	0.024[0.010, 0.036]	0.078	6543.895	6827.888	0.005	−0.007
	Scalar	90.786(62)	0.960	0.953	0.026[0.013, 0.036]	0.075	6550.980	6792.899	−0.008	0.002

*RPQ* the Reactive- proactive aggression questionnaire, *HADS* the hospital anxiety and depression scale, *CES-D* the center for epidemiologic studies depression scale, *CA* the cyberbullying questionnaire, *CFI* the comparative fit index, *TLI* the tucker–lewis index, *RMSEA* the root mean square error of approximation, *SRMR* the standardized root mean square residual, *AIC* the akaike information criterion, *BIC* the bayesian information criterion

Moreover, the ICC for reactive aggression was 0.315, suggesting that 31.5% of the variance was due to between-person differences and 68.5% was due to within-person fluctuations. For proactive aggression, the ICC was 0.444, which indicated that 44.4% of the variance in proactive aggression was explained by differences between adolescents, while the remaining 55.6% was explained by fluctuations within adolescents over time. For depressive symptoms and anxious symptoms, the ICC values were 0.460 and 0.263, respectively. Thus, 54.0% and 73.7% of the variances in those variables in the study were explained by fluctuations within adolescents over time. Furthermore, the intra-individual differences (ICC = 0.453) explained 54.7% of the variance in cyber aggression.

### RI-CLPM Results

**Fig. 1:**
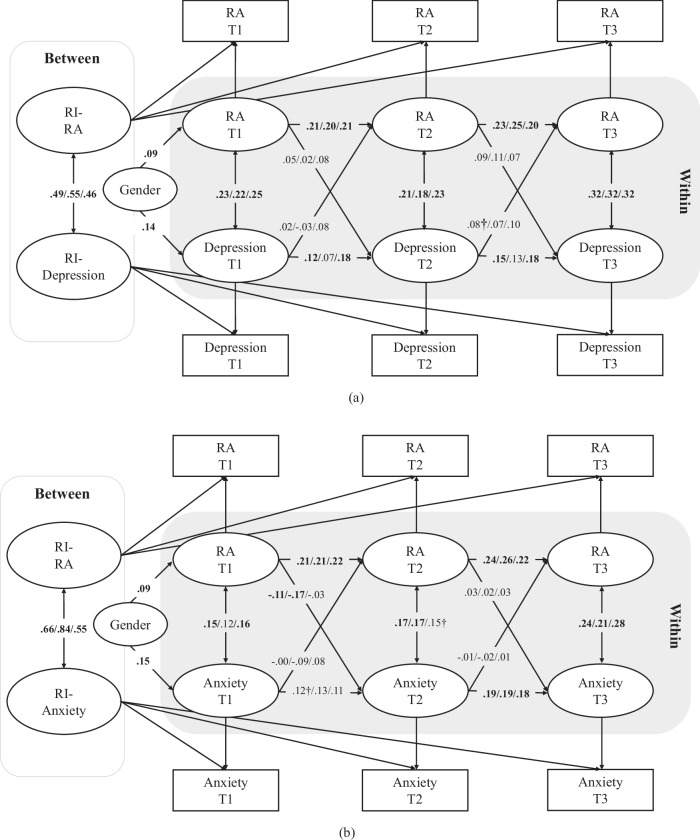
RI-CLPM for reactive aggression and depression in the whole / male / female sample. RA reactive aggression. The values in bold represent statistically significant paths at least at *p* < 0.05; †*p* < 0.10

**Fig. 2:**
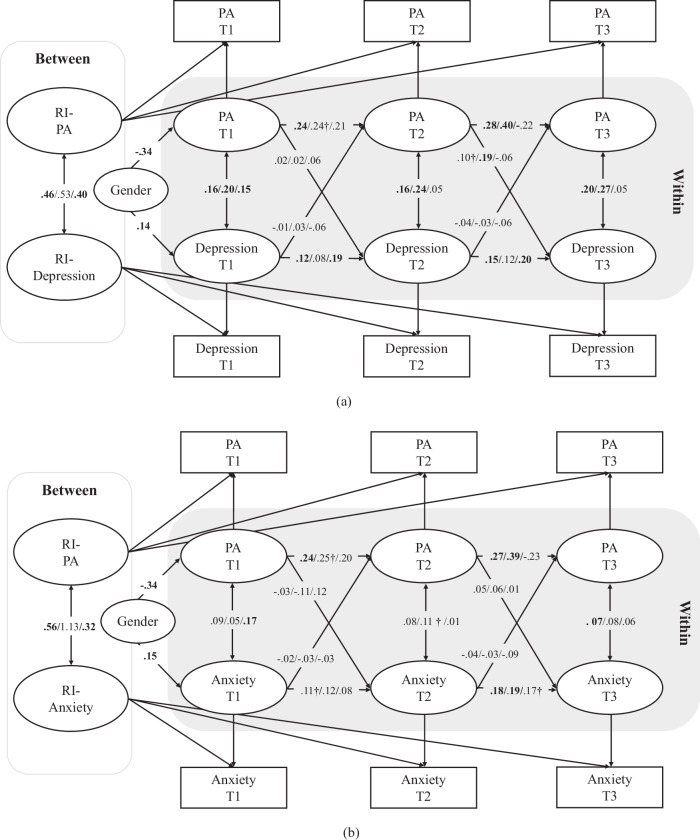
RI-CLPM for proactive aggression and depression in the overall / male / female sample. PA proactive aggression. The values in bold represent statistically significant paths at least at *p* < 0.05; †*p* < 0.10

**Fig. 3:**
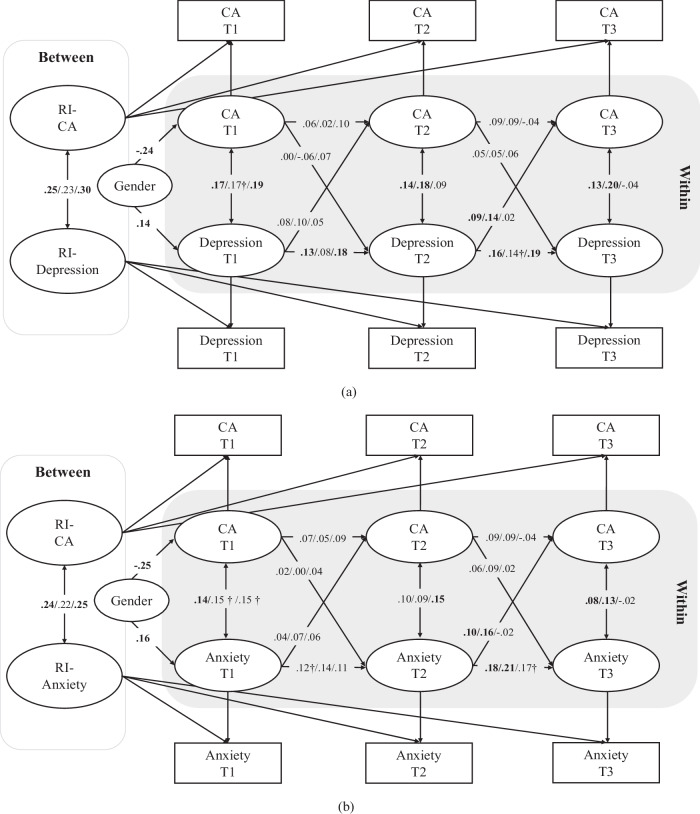
RI-CLPM for cyberbullying and depression in the overall / male / female sample. CA cyber-aggression. The values in bold represent statistically significant paths at least at *p* < 0.05; †*p* < 0.10

Overall, all of the RI-CLPMs exhibited good model fits (see Table 3), with the exceptions of M5 for the full sample and M6 for boys and the full sample, both of which showed slightly poor fits (i.e., TLI values smaller than 0.90). At the between-person level, the adolescents exhibiting higher levels of reactive aggression had greater levels of depressive symptoms (*β* = 0.49, *p* < 0.001; Fig. 1a) and also of anxious symptoms (*β* = 0.66, *p* < 0.001; Fig. 1b). The adolescents exhibiting higher levels of proactive aggression also tended to show both more depressive symptoms (*β* = 0.46, *p* < 0.01; Fig. 2a) and more anxious symptoms (*β* = 0.56, *p* < 0.01; Fig. 2b) than their peers did. Finally, the adolescents exhibiting higher levels of cyber aggression also had higher levels of depressive symptoms (*β* = 0.25, *p* < 0.01; Fig. 3a) and anxious symptoms (*β* = 0.24, *p* < 0.01; Fig. 3b).

**Table 3:** Model fit indices for all models in the full (*n* = 1422)/male (*n* = 698)/female (*n* = 724) sample

	CFI full/male/female sample	TLI full/male/female sample	RMSEA full/male/female sample	SRMR full/male/female sample
M1: RA-DE	0.999/1.000/1.000	0.998/1.027/1.016	0.011/<0.001/<0.001	0.009/0.003/0.002
M2: RA-Anx	0.999/1.000/0.999	0.998/0.993/0.989	0.011/0.019/0.029	0.010/0.008/0.009
M3: PA-DE	0.998/1.000/1.000	0.990/1.013/1.003	0.016/<0.001/<0.001	0.010/0.007/0.007
M4: PA-Anx	1.000/1.000/1.000	1.012/1.019/1.037	<0.001/<0.001/<0.001	0.007/0.006/0.001
M5: CA-DE	0.967/1.000/1.000	0.863/1.043/1.016	0.053/<0.001/<0.001	0.021/0.005/0.007
M6: CA-Anx	0.973/0.991/0.998	0.888/0.859/0.967	0.046/0.051/0.031	0.019/0.013/0.009

*RA* reactive aggression, *DE* depression, *PA* proactive aggression, *CA* cyber aggression, *Anx* anxiety

At the within-person level, reactive aggression at T1 predicted a decrease in anxious symptoms at T2 (*β* = –0.11, *p* = 0.046), but this relationship was significant only among the boys (*β* = –0.17, *p* = 0.017, Fig. 1b). No significant cross-lagged effects were found between proactive aggression and anxious symptoms in the full sample or the gender-stratified sample (Fig. 2b). A significant positive within-person association was noted between proactive aggression at T2 and depressive symptoms at T3 (*β* = 0.19, *p* = 0.015) among the boys, but this effect was only marginally significant in the full sample (*β* = 0.10, *p* = 0.095, Fig. 2a). Overall, Hypothesis 1 was partially supported.

Hypothesis 2 was also partially supported. As is shown in Fig. 1a, depressive symptoms predicted higher reactive aggression one year later (i.e., from T2 to T3) in the full sample, although this result was only marginally statistically significant (*β* = 0.08, *p* = 0.054). Interestingly, in gender-specific analyses, this cross-lagged effect was not significant for either the boys or the girls (*p* > 0.05). Depressive symptoms at T2 predicted a within-person increase in cyber aggression at T3 in the full sample (*β* = 0.09, *p* = 0.043), but this relationship was significant only among the boys (*β* = 0.14, *p* = 0.027) in the gender-stratified analyses (Fig. 3a). In addition, there was a positive within-person relationship between anxious symptoms at T2 and cyber aggression at T3 (*β* = 0.10, *p* = 0.024), and this effect, too, was significant only among the boys (*β* = 0.16, *p* = 0.006; Fig. 3b).

The results also showed stability in the development of depressive symptoms, especially among the girls. As is shown in Fig. 1a, positive relationships were noted between depressive symptoms at T1 and at T2 in the full sample (*β* = 0.12, *p* < 0.05), although this was significant only among the girls (*β* = 0.18, *p* < 0.05). Similar relationships were noted between depressive symptoms at T2 and at T3 in the full sample (*β* = 0.15, *p* < 0.05), which was again significant only among the girls (*β* = 0.18, *p* < 0.05). Figure 2a shows positive relationships between depressive symptoms at T1 and at T2 in the full sample (*β* = 0.12, *p* < 0.05), which was significant only among the girls (*β* = 0.19, *p* < 0.05), and between depressive symptoms at T2 and at T3 in the full sample (*β* = 0.15, *p* < 0.05), which also was significant only among the girls (*β* = 0.20, *p* < 0.05). Finally, Fig. 3a shows that there were also positive relationships between depressive symptoms at T1 and at T2 in the full sample (*β* = 0.13, *p* < 0.05), which was only significant among the girls (*β* = 0.18, *p* < 0.05), and between depressive symptoms at T2 and at T3 in the full sample (*β* = 0.16, *p* < 0.05), which again was only significant among the girls (*β* = 0.19, *p* < 0.05).

### Multiple-group RI-CLPM Results

This study performed multiple-group RI-CLPMs to test the moderating effect of gender (Mulder & Hamaker, 2021). First, multiple-group RI-CLPMs without constraints over the different gender groups were fitted, and the results showed a good model fit for all of the test models. Then, the lagged parameters were constrained to be equal over time, and the studied models showed acceptable to good model fits. Table 4 presents the results. In addition, the chi-square difference tests for Models M1, “reactive aggression–depressive symptoms;” M2, “reactive aggression–anxious symptoms;” M5, “cyber aggression–depressive symptoms;” and M6, “cyber aggression–anxious symptoms” were Δ*χ*^2^(8) = 3.672 (*p* = 0.891), Δ*χ*^2^(8) = 5.200 (*p* = 0.712), Δ*χ*^2^(8) = 12.929 (*p* = 0.135), and Δ*χ*^2^(8) = 9.293 (*p* = 0.262), respectively, thus implying that males and females appear to have the same lagged effects (Mulder & Hamaker, 2021). However, Model M3 “proactive aggression–depressive symptoms” and Model M4 “proactive aggression–anxious symptoms” showed significant chi-square differences, suggesting that there may be different lagged effects between males and females. Further analyses showed that the prediction from proactive aggression at T2 to depressive symptoms at T3 was significantly stronger for males than for females (Wald test = 4.172, *p* = 0.041). In comparison, the prediction from proactive aggression at T1 to anxious symptoms at T2 was marginally stronger for females (Wald test = 3.765, *p* = 0.052). Moreover, the predictions from proactive aggression at Time 2 to proactive aggression at Time 3 in these two models were stronger for males (Wald test = 7.864, *p* = 0.005; Wald test = 7.548, *p* = 0.006).

**Table 4:** Multiple group RICLPM

		*χ*^*2*^(*df*)	RMSEA	CFI	SRMR	Δχ^2^(*df*)	S-B χ^2^(*df*)	*p*
M1: RA-DEP	Basic model	0.206(2)	0.000[0.000, 0.032]	1.000	0.002	3.672(8)	3.601(8)	0.891
	Constraint model	3.878(10)	0.000[0.000, 0.000]	1.000	0.013			
M2: RA-ANX	Basic model	2.844(2)	0.024[0.000, 0.082]	0.999	0.009	5.200(8)	5.421(8)	0.712
	Constraint model	8.044(10)	0.000[0.000, 0.034]	1.000	0.015			
M3: PA-DEP	Basic model	1.686(2)	0.000[0.000, 0.071]	1.000	0.007	35.391(8)	33.129(8)	<0.001
	Constraint model	37.077(10)	0.062[0.041, 0.084]	0.960	0.037			
M4: PA-ANX	Basic model	0.593(2)	0.000[0.000, 0.052]	1.000	0.004	38.700(8)	36.388(8)	<0.001
	Constraint model	39.293(10)	0.064[0.044, 0.086]	0.956	0.037			
M5: CA-DEP	Basic model	1.082(2)	0.000[0.000, 0.062]	1.000	0.006	12.929(8)	12.384(8)	0.135
	Constraint model	14.011(10)	0.024[0.000, 0.050]	0.993	0.028			
M6: CA-ANX	Basic model	4.638(2)	0.043[0.000, 0.096]	0.995	0.011	9.293(8)	10.045(8)	0.262
	Constraint model	13.931(10)	0.024[0.000, 0.050]	0.992	0.029			

*RMSEA* the root mean square error of approximation, *CFI* the comparative fit index, *SRMR* the standardized root mean square residual, *S-B χ*^*2*^ satorra-bentler scaled χ^2^ test, *RA* reactive aggression, *DE* depression, *PA* proactive aggression, *CA* cyber-aggression, *Anx* anxiety

## Discussion

Research has established clear links between aggression and depressive or anxious symptoms, but it has yet to fully explore how these interactions evolve over time, particularly by distinguishing within-person and between-person effects. This longitudinal study has examined the interplays between depressive symptoms and traditional aggression and cyber aggression, and between anxious symptoms and the two types of aggression, using RI-CLPMs. Informed by the frustration-aggression theory and the failure model, this study constructed six RI-CLPMs to explore the longitudinal relationships between aggressive behaviors (reactive, proactive, and cyber aggression) and two mental health problems (depressive and anxious symptoms) among Chinese adolescents. The use of RI-CLPMs was vital for elucidating the intersecting dynamics among the variables through a separation of between-person and within-person effects (Hamaker et al., 2015). The current results supported the associations between each type of aggressive behavior and the mental health conditions at the between-person level, which suggest that, at the population level, adolescents with higher levels of aggression are at a higher risk of developing depression and anxiety than those with lower levels of aggression are––thus supporting previous evidence on the associations between aggression and both depression and anxiety (e.g., Huesmann et al., 2019; Zhang et al., 2020). Moreover, the results from the within-person level analyses were relatively mixed in this study, so the within-person analysis results will be elaborated upon in the following discussion.

### Persistence of Adolescents’ Mental Health Conditions and Aggression

In addition to the findings delineating the cross-lagged paths, the autoregressive results also highlighted the developmental trajectories of adolescent aggression and mental health conditions at the within-person level. Generally, all of the autoregressive paths, except for that for cyber aggression, were statistically significant for the entire sample across the three time points, thus indicating that reactive and proactive aggression and depressive and anxiety appeared to be stable over time and pointing to the persistence of these misconducts and disorders among adolescents. It is inferred that because cyber aggression can take place in a more casual and unconscious way, cyber aggressors may not always be aware of or interpret their behaviors as actually being cyber aggression.

### Bidirectional Relationships between Adolescents’ Mental Health Conditions and Aggression

According to the frustration-aggression theory, the presence of depression and anxiety can trigger aggressive behaviors, given that both depression and anxiety are typical outcomes of persistently frustrating events and that individuals may be motivated by frustration to restore a sense of significance or importance through aggressive behaviors. However, this study failed to find stable, significant cross-lagged effects between reactive/proactive aggression and depressive symptoms over time in the full sample at the within-person level, which is partially consistent with the findings of another recent study (Yang et al., 2024a). Early adolescents may have already achieved the critical sociodevelopmental milestones that facilitate the co-occurrence of depression and aggression without a direct influence between the two (Yang et al., 2024a). In addition, from a methodological perspective, the use of an RI-CLPM has been noted to increase the likelihood of non-significant effects, which could be attributed to the complexity of the method and the introduction of random intercepts that help control for confounding factors. Specifically, the changes in outcome levels have been found to tend to be minimal due to the significant stability of most psychological constructs over time. In addition, adjusting for stability effects (i.e., previous levels of constructs) may eliminate a big portion of the outcome variance that is common with other predictors (Adachi & Willoughby, 2015). Consequently, eliminating between-person variance may also reduce some of the variance in the outcomes. Nevertheless, this study still found evidence of a weak predictive relationship between aggression and mental health.

This study further found that depressive symptoms at T2 positively predicted reactive aggression and cyber aggression at T3 at the within-person level (marginally significant, at the *p* < 0.1 level for depressive symptoms–reactive aggression). These findings echo previous longitudinal evidence suggesting that depression can be expressed through aggressive behaviors (e.g., Kofler et al., 2011; Ozkan et al., 2019). Although depression may not necessarily increase the likelihood of impulsive and inhibitory aggression, which in turn leads to violence and crime, the attention deficits and interpersonal issues caused by depression can indeed contribute to violent tendencies and criminal behavior (Remster, 2014). Negative emotions may be expressed through antisocial actions for relieving stress, and thus depression is a risk factor for future delinquency (Ozkan et al., 2019). However, this finding warrants cautious interpretation and needs to be replicated in future research.

Furthermore, anxious symptoms were found to positively predict only later cyber aggression and were not significantly associated with the other two types of in-person aggression. In other words, whereas depressive symptoms could lead an adolescent to engage in both online and offline aggressive behaviors, anxious symptoms catalyzed only cyber aggression. This result can be interpreted through the lens of the frustration-aggression theory as follows. First, assuming that aggressors in online and in-person settings are confronted with approximately identical levels of obstacles that prevent them from actualizing their aggression (i.e., external constraints to aggression are held constant), perhaps the individual’s motivation to restore his/her significance is reflected more strongly through depression than through anxiety. Second, assuming that depression and anxiety generate approximately identical levels of motivation to behave aggressively (i.e., that internal constraints to aggression are held constant), anxiety may lead potential aggressors to anticipate higher levels of penalties for in-person aggression, whereas they may not perceive the consequences of cyber aggression to be equally punitive, given its anonymous nature. Moreover, visible aggression in the Chinese culture is held to reflect a “loss of face” (Xu et al., 2016), thus making cyberbullying likely to be considered a more appropriate way for individuals to vent negative emotions. In summary, the study’s findings lend partial support to the frustration-aggression theory, and future studies should take the above-mentioned factors into consideration.

In regard to changes in adolescents’ mental health conditions over time after they engage in aggressive behaviors, the study’s evidence partially contradicts the stated hypothesis. According to the failure model, aggressive behaviors may result in social rejection and other social penalties that in turn may lead aggressors to suffer higher levels of mental problems (especially depression). The analyses showed that proactive-aggressive behaviors, but not reactive-aggressive behaviors, appeared to cause a later increase in levels of depressive symptoms––specifically, the adolescents’ proactive aggression at T2 positively predicted depressive symptoms at T3 (*p* < 0.1). Moreover, reactive-aggressive adolescents in this sample were found to display lower levels of anxious symptoms than others did, which was counterintuitive.

These results appear to reflect that the causality between aggression and social rejection may be context-dependent. The failure model presumes that the denouncement of aggressive behaviors is a ubiquitous social norm which is independent of social contexts. However, it is anticipated that the presence of several contextual or incidental factors may be able to interrupt that path. First, it is likely that some societies are more tolerant than others of adolescent aggression (e.g., those that deem aggression as a demonstration of masculinity or independence). Furthermore, at the meso level, within social institutions such as the family, school, and/or neighborhood, it is reasonable to expect that adolescents may internalize aggression as a norm. For example, a cross-sectional study found that high scores on proactive-aggressive behaviors were associated with low scores on anxious symptoms among children whose parents adopted a highly authoritative parenting style (Pederson et al., 2016). Authoritative parents usually provide better social support to their children, leaving the children more confident of their behaviors and thus possibly legitimizing aggressive behaviors. In summary, the causal relationship from aggression to later mental health conditions is subject to the actual social context within which the individual interacts. In that light, contextual factors emerging from both the macro and meso levels should be further examined to determine the context-dependent relationships between aggressive behaviors and mental health conditions.

### Gender Differences in the Relationships between Aggression and Mental Health Conditions

The relationships between aggression and mental health conditions appear to vary across genders. In this study, when the RI-CLPM models were tested separately in male and female groups, the longitudinal relationships between different forms of aggression and mental health conditions seemed to be stronger in males than in females (i.e., the path from reactive aggression at T1 to anxious symptoms at T2; the path from proactive aggression at T2 to depressive symptoms at T3; the path from depressive symptoms at T2 to cyber aggression at T3; and the path from anxious symptoms at T2 to cyber aggression at T3). These findings suggest that the relationships between aggression and mental health may be robust within Chinese male adolescents, but that robustness does not exhibit cross-temporal stability.

Further analyses of multiple-group RI-CLPMs showed that there were gender differences in the relationships between proactive aggression and both depressive and anxious symptoms. Specifically, the positive association between proactive aggression at T2 and depressive symptoms at T3 in male adolescents was stronger than that in female groups. Previous empirical evidence also showed that boys were more likely than girls to exhibit both internalizing and externalizing problems concurrently, from childhood to adolescence (e.g., Shi et al., 2020). Interestingly, however, although in general boys seem to show more aggressive behaviors than girls do, in the context of Chinese culture aggression is not allowed and tends to be suppressed and even severely punished, with a cultural emphasis on collectivism and harmony––and that pattern may lead individuals to negative self-evaluation and self-criticism, which in turn may trigger depression (Chen & French, 2008; Yang et al., 2024b). In addition, this study found that the female group showed a slightly stronger, marginally significant association between proactive aggression at T1 and anxious symptoms at T2 compared with the male adolescents. It is widely believed that through social norms, girls encounter more restraints and prohibitions than boys do regarding aggressive behaviors (Eagly & Steffen, 1986). In that event, compared with boys, girls may experience greater guilt and anxiety after engaging in aggression. Indeed, girls may have heightened concerns about the potential harm their aggressive actions could cause, and they may even fear retaliation (e.g., Archer, 2004). However, in this study the discrepancy between the two gender groups was only marginally significant, thus suggesting that additional research is warranted on how gender affects the relationship between proactive aggression and anxiety.

### Limitations and Implications

Although the findings of this study shed important light on the longitudinal relationships between adolescent aggression and mental health conditions, the study also had limitations that must be acknowledged. First, the cross-lagged effects from the mental health conditions to the three types of aggression were small, and some paths only showed significance at the *p* = 0.10 level, implying that this risk appears to be minor over the three-year course of adolescence. Therefore, these findings should be interpreted with caution. In addition, the nature of mental health conditions and their association with different types of aggression need to be investigated over a longer time span and with repeated measurements made at shorter intervals. Moreover, this study only investigated the reciprocal relationships between reactive and proactive aggression and depressive and anxious symptoms, while other forms of aggression (e.g., relational aggression or overt aggression), and other mental health problems, such as social withdrawal, warrant further measurement in future research. Nevertheless, the current study can still provide valuable preliminary insights into the longitudinal links between different forms of aggressive behavior and mental health among Chinese adolescents––an important topic that has been relatively underexplored in the literature.

Second, self-reported measures can be biased by socially desirable responses and common method variance. To overcome these drawbacks, future studies should adopt multiple data-collection strategies, including multiple-informant formats (e.g., caregiver- or teacher-reported formats) and interviews. Furthermore, although this study adopted the random sampling approach, the interdependence of observations arising from this clustered data structure was not explicitly modeled. Future investigations could be strengthened by incorporating multilevel analytic techniques. Finally, the sample in this study was not nationally representative. Given that the sample of adolescents was recruited only from a particular province of mainland China, the findings may not be generalizable to adolescents in other regions of the country, nor to other societies.

Nonetheless, this is the first study undertaken to examine the longitudinal relationships between different types of aggression (i.e., reactive, proactive, and cyber aggression) and mental health problems (i.e., depressive and anxious symptoms) at the between-person and within-person levels in Chinese youth. By controlling for fluctuating levels of relevant constructs, this study found that adolescents with higher levels of mental health problems tended to display higher than expected levels of cyber aggression, and conversely, adolescents who experienced higher levels of proactive aggression than typical also tended to experience mental health problems. Therefore, the findings are valuable for understanding how both depressive and anxious symptoms and the different types of aggression, develop and affect each other in adolescence. Specifically, the relationships between various forms of aggression and depressive or anxious symptoms can influence the theorization of developmental processes in Chinese youth in terms of the temporal sequence of mental health conditions and aggression. Additional longitudinal investigations into the relationships between internalizing problems and different types of aggression, using a large sample, could help identify important specific periods of vulnerability in adolescent development. Because mental health problems can significantly contribute to the development of aggressive behaviors, especially cyber aggression (Chen et al., 2017; Zhang et al., 2020), educators should focus on mitigating the onset of depression and/or anxiety in adolescence by understanding students’ emotional states and offering a more relaxed environment that enhances the students’ autonomy.

Meanwhile, to improve treatment effectiveness, therapeutic approaches could be tailored on the basis of the dynamics between different types of aggression and specific mental health problems. Schools and community organizations could establish supportive systems and programs aimed at reducing both aggressive behaviors and mental health issues, to help foster young people’s resilience and emotion-regulation skills. In addition, the gender differences uncovered in the relationships between proactive aggression and mental health problems suggest that interventions should incorporate a gender-sensitive approach and should address the unique risk factors and coping strategies associated with each gender. For instance, interventions targeting boys could enhance their emotional awareness and promote positive outlets for anger. An enhanced understanding of the gender-specific mechanisms in those relationships could inform more effective programs for fostering mental health and reducing aggression.

## Conclusion

Although it is evident that aggression is closely correlated with depressive and anxious symptoms, few studies have examined the dynamic interplay between multiple forms of aggression and mental health conditions over time by distinguishing the within-person and between-person effects. To address the theoretical, methodological, and empirical gaps in the existing literature, this study adopted RI-CLPM models to investigate the longitudinal associations between different types of aggression (i.e., reactive, proactive, and cyber aggression) and symptoms of depression and anxiety in a large sample of Chinese adolescents. In line with the frustration-aggression theory and the failure model, this study found that both depressive and anxious symptoms predicted cyber aggression across certain time points. Moreover, reactive aggression predicted reduced levels of anxious symptoms, and proactive aggression had a longitudinal, positive effect on depressive symptoms. Knowledge of such longitudinal, bidirectional relationships between mental health conditions and aggression in adolescents can help educators and clinicians develop targeted, early prevention and intervention strategies that may ultimately curb the progression of mental health problems and aggressive behaviors among youth.
